# ADULT DAY SERVICES DATA COLLECTION AND OUTCOMES: A NATIONAL STRATEGY REALIZED

**DOI:** 10.1093/geroni/igad104.0101

**Published:** 2023-12-21

**Authors:** William Zagorski

**Affiliations:** American Senior Care Centers, Inc., Nashville, Tennessee, United States

## Abstract

The National Adult Day Services Association (NADSA), in its commitment to advance the development and recognition of adult day services across the United States, has defined a comprehensive set of data points and uniform outcome measures to be utilized throughout the Adult Day Services (ADS) industry. NADSA represents more than 500 unique Adult Day Service Centers (ADSCs) across the country. This project has culminated in the opening of a single national secure data entry portal and repository for the collection of this data free to use for adult day service providers across the country. Prior to its full release in January 2023, the beta testing phase allowed for the utilization of this platform across nearly a dozen states, more than 20 ADSCs and more than 500 ADS participants in a variety of settings. As of January 2023, the system is now available to more than 500 ADSCs serving more than 12,000 participants. This session will describe the data points and outcome measures as well as the uniform tools to be utilized for collection and introduce the data portal to the greater academic community. This longitudinal data will be available to NADSA academic partners to provide greater avenues of research into the benefits of adult day services use to participants, caregivers, payors, and the community.

